# Small-scale land-use variability affects *Anopheles* spp. distribution and concomitant *Plasmodium* infection in humans and mosquito vectors in southeastern Madagascar

**DOI:** 10.1186/s12936-016-1164-2

**Published:** 2016-02-24

**Authors:** Sarah Zohdy, Kristin Derfus, Emily G. Headrick, Mbolatiana Tovo Andrianjafy, Patricia C. Wright, Thomas R. Gillespie

**Affiliations:** Department of Environmental Sciences and Program in Population Biology, Ecology, and Evolution, Emory University, 400 Dowman Drive, Suite E510, Atlanta, GA 30322 USA; Department of Environmental Health, Rollins School of Public Health, Emory University, 1518 Clifton Road NE, Atlanta, GA 30322 USA; Nell Hodgson Woodruff School of Nursing, Emory University, 520 Clifton Road NE, Atlanta, GA 30322 USA; Department of Entomology, University of Antananarivo, Antananarivo, Madagascar; Centre ValBio, Ranomafana Ifanadiana 312, BP 33, Ranomafana, Madagascar; Department of Anthropology, Stony Brook University, Stony Brook, NY 11794-4364 USA

**Keywords:** Disturbance ecology, Ifanadiana District, *Plasmodiumfalciparum*, *Plasmodiumvivax*, Bed net

## Abstract

**Background:**

Deforestation and land-use change have the potential to alter human exposure to malaria. A large percentage of Madagascar’s original forest cover has been lost to slash-and-burn agriculture, and malaria is one of the top causes of mortality on the island. In this study, the influence of land-use on the distribution of *Plasmodium* vectors and concomitant *Plasmodium* infection in humans and mosquito vectors was examined in the southeastern rainforests of Madagascar.

**Methods:**

From June to August 2013, health assessments were conducted on individuals living in sixty randomly selected households in six villages bordering Ranomafana National Park. Humans were screened for malaria using species-specific rapid diagnostic tests (RDTs), and surveyed about insecticide-treated bed net (ITN) usage. Concurrently, mosquitoes were captured in villages and associated forest and agricultural sites. All captured female *Anopheline* mosquitoes were screened for *Plasmodium* spp. using a circumsporozoite enzyme-linked immunosorbent assay (csELISA).

**Results:**

*Anopheles* spp. dominated the mosquito communities of agricultural and village land-use sites, accounting for 41.4 and 31.4 % of mosquitoes captured respectively, whereas *Anopheles* spp. accounted for only 1.6 % of mosquitoes captured from forest sites. Interestingly, most *Anopheles* spp. (67.7 %) were captured in agricultural sites in close proximity to animal pens, and 90.8 % of *Anopheles* mosquitoes captured in agricultural sites were known vectors of malaria. Three *Anopheline* mosquitoes (0.7 %) were positive for malaria (*Plasmodium vivax*-210) and all positive mosquitoes were collected from agricultural or village land-use sites. Ten humans (3.7 %) tested were positive for *P. falciparum*, and 23.3 % of those surveyed reported never sleeping under ITNs.

**Conclusions:**

This study presents the first report of malaria surveillance in humans and the environment in southeastern Madagascar. These findings suggest that even during the winter, malaria species are present in both humans and mosquitoes; with *P. falciparum* found in humans, and evidence of *P. vivax*-210 in mosquito vectors. The presence of *P. vivax* in resident vectors, but not humans may relate to the high incidence of humans lacking the Duffy protein. The majority of mosquito vectors were found in agricultural land-use sites, in particular near livestock pens. These findings have the potential to inform and improve targeted malaria control and prevention strategies in the region.

**Electronic supplementary material:**

The online version of this article (doi:10.1186/s12936-016-1164-2) contains supplementary material, which is available to authorized users.

## Background

Madagascar is a malaria-endemic country where malaria ranks fourth among causes of reported mortality. In 2011, malaria was the second leading cause of death among children under 5 years [1]. While malaria epidemiology varies considerably in different regions of the country, with perennial transmission in the southeastern regions of Madagascar [1], specific transmission seasons in the lowland areas in the northwest, and unstable seasonal transmission in the highland (central) and semi-desert areas (southwest) areas of the country, the entire population is considered to be at risk for the disease [1].

Of the five *Plasmodium* species capable of infecting humans with malaria, four are present in Madagascar [2], with *Plasmodium falciparum* as the most prevalent species, followed by *P. vivax* [2]. According to [2], the expected vector species of *Anopheles* in the region are *Anopheles funestus*, *An. gambiae*, and *An. arabiensis*, and more recently *An. coustani* [3, 4]. Although increased financing for malaria treatment and mosquito control with insecticides has successfully decreased malaria rates in Madagascar [1], addressing environmental determinants of disease could provide additional ways of controlling malaria, including environmental management practices, such as vegetation clearance, draining swamps and modification of river boundaries [5, 6], that would increase the efficiency of programmes [5].

Madagascar is a unique island environment with a high level of animal and plant endemism; however, to date over 90 % of the original forests on the island have been lost due to slash-and-burn agricultural practices associated with human population growth, which have led to rapid conversion of forest to land used for rice production [7]. In other parts of the world, this sort of deforestation has been linked to deleterious effects on environmental conditions capable of enhancing opportunities for human pathogens [8, 9]; and the loss of ecosystem resources through land-use conversion also has the potential to increase the risk of human malaria infection by altering nutrient enrichment and watershed dynamics on a local scale, creating an abundance of new *Anopheles* mosquito breeding habitats [8–11], for example, through irrigation, which often leads to an increase in mosquitoes and malaria prevalence [5].

In an effort to protect the original forests from land-use conversion, several national parks, such as Ranomafana National Park (RNP) in the southeastern rainforests of Madagascar, have been established [12]. Despite its protected status, RNP is threatened by accelerating forest conversion due to slash-and-burn agricultural practices. More than 85 % of the estimated 54,000 people in the region immediately surrounding RNP rely primarily on subsistence agriculture and the slash-and-burn practice remains a cultural norm [13]. Considering the importance of malaria as a cause of mortality in Madagascar [2], an understanding of the relationship between land-use conversion and malaria vector distribution and concomitant *Plasmodium* infection in humans and mosquito vectors on a local scale is necessary. Consequently, this study was conducted to: (1) determine the distribution of *Plasmodium* vectors and concomitant *Plasmodium*-status of vectors relative to small-scale variability in land-use patterns, and (2) examine demographic and behavioural associations with human *Plasmodium* spp. infection in this system.

## Methods

### Ethics statement

All research protocols were presented to and approved by the USDA and the Government of Madagascar. The United States Veterinary Permit for Importation and Transportation of Controlled Materials and Organisms and Vectors (Permit # 107234) was used. Although initially reviewed for approval by the Emory University’s Institutional Review Board, this work was subsequently determined to be ‘public health practice, with the goal of benefit to people in the region’ and therefore exempt from further human subjects review. In Madagascar, the IRB protocol was reviewed and approved by the Director of Health in the Fianarantsoa district. Both verbal and written consent were obtained for all participants 18 years and older. Verbal and written assent were obtained for those 10–17 years of age, and parents of children under the age of 10 acted as proxies for their children for the individual surveys.

### Study site

This work was conducted in six rural villages bordering Ranomafana National Park (RNP) (21°02′–21°25′S, 47°18′–47°37′E) in the Ifanadiana District of Madagascar. RNP is a continuous humid tropical forest with natural vegetation ranging from montane cloud forest to lowland rainforest following an altitudinal gradient from 1513 to 600 metres above sea level [10].

### Study procedures

Villages were defined as communities with at least 10 homes within 15 metres from one another, and are within ≤3 km of the boundaries of Ranomafana National Park. Six villages were randomly selected, and in each of the villages, 10 households were randomly selected to participate in the survey, malaria testing, and health assessment components of the study, for a total of sixty households. All age groups and sexes were eligible for testing. After completing informed consent forms, individuals were given health assessments measuring height, weight, temperature, blood pressure, and heart rate (Additional file 1). Individuals also received a malaria RDT (First Response Malaria Ag Combo Kits, Premier Med Corps, Nani Daman, India) to determine *Plasmodium* spp. infection. These tests are optimized to detect four malaria species: *P. vivax*, *P. ovale*, and *P. malariae* through a PAN test line specific to lactate dehydrogenase (pLDH), and *P. falciparum* through a Histidine-Rich Protein 2 (HRP2) specific to the species. Individuals testing positive for malaria were provided with recommended artemisinin combined therapy and advised to seek follow up care should symptoms persist. Individuals were also surveyed about insecticide-treated net (ITN) ownership and usage (Additional file 2).

**Fig. 1:**
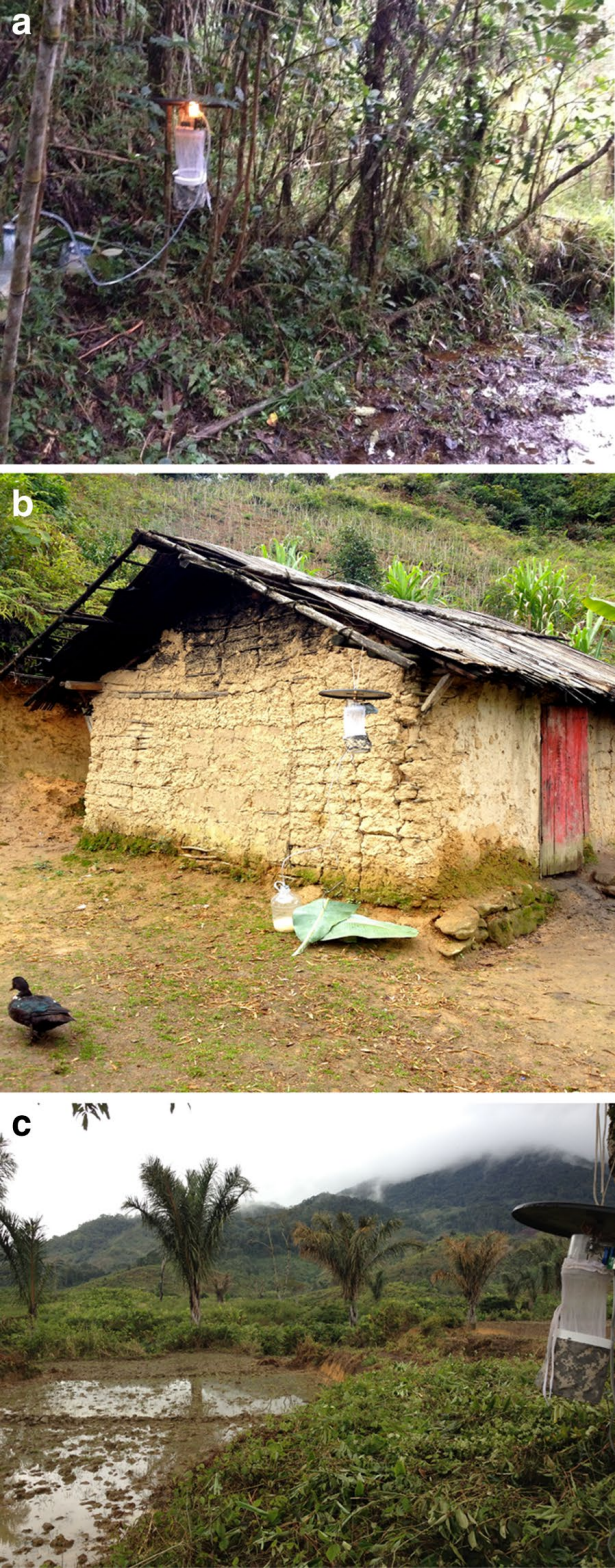
Examples of trapping sites in and around six villages near Ranomafana National Park, Madagascar. **a** Forest-trapping site. **b** Village-trapping site. **c** Agricultural-trapping site

Mosquitoes were trapped in the six villages and their associated agricultural and forest sites using two traps in each site. The two traps in the villages did not correspond to the households randomly selected for survey administration, but rather were placed on opposite ends of the village (Table 1) outside of households. Six traps were set per night for three consecutive nights in each village (four consecutive nights, battery permitting). Mosquitoes were trapped for a total of eighteen trapping nights at thirty-six trapping sites. Forest and agricultural sites were all <1 km from the village center. In most cases, land-use sites were contiguous (Table 1; Fig. 1). Agricultural sites were all former ‘tavy’ (slash-and-burn agriculture) sites where the forest has been burned and converted into rice paddies that are irrigated by rainfall. Forest sites included primary and secondary forest within national park boundaries. These sites had no human inhabitants and ranged from little (forest trails, trails to a few homes, etc.) to no daily human overlap (Fig. 1a).

**Table 1:** Geographic coordinates and dates of sampling for mosquito trap sites in six villages near Ranomafana National Park, Madagascar

	Trap	Location	Elevation (m)
Ambatolahy (6/17/13–6/21/13)	Forest	S 21°15′05.6″ E 047°25′21.6″	1011
	Forest w/odor	S 21°15′07.5″ E 047°25′23.3″	930
	Village	S 21°14′57.3″ E 047°25′48.2″	865
	Village w/odor	S 21°14′58.8″ E 047°25′47.3″	856
	Agriculture	S 21°15′00.6″ E 047°25′48.1″	872
	Agriculture w/odor	S 21°15′02.1″ E 047°25′47.7″	876
Vohiparara (6/30/13–7/4/13)	Forest	S 21°14′17.8″ E 047°23′41.4″	1129
	Forest w/odor	S 21°14′08.5″ E 047°23′46.9″	1129
	Village	S 21°14′20.7″ E 047°22′53.0″	1133
	Village w/odor	S 21°14′20.1″ E 047°22′54.7″	1136
	Agriculture	S 21°14′11.4″ E 047°23′07.0″	1129
	Agriculture w/odor	S 21°14′22.5″ E 047°22′58.2″	1131
Ambodiaviavy (7/7/13–7/10/13)	Forest	S 21°15′26.4″ E 047°28′34.1″ ± 10 m	744
	Forest w/odor	S 21°15′23.4″ E 047°28′34.1″ ± 10 m	781
	Village	S 21°15′48.8″ E 047°29′06.0″	640
	Village w/odor	S 21°15′50.8″ E 047°29′05.7″	642
	Agriculture	S 21°15′45.6″ E 047°29′03.3″	623
	Agriculture w/odor	S 21°15′50.4″ E 047°29′09.2″	619
Menarano (7/20/13–7/24/13)	Forest	S 21°17′27.9″ E 047°27′16.3″	834
	Forest w/odor	S 21°17′27.2″ E 047°27′20.2″	812
	Village	S 21°17′26.3″ E 047°28′07.9″	716
	Village w/odor	S 21°17′25.3″ E 047°28′07.2″	715
	Agriculture	S 21°17′34.9″ E 047°28′06.5″	686
	Agriculture w/odor	S 21°17′32.2″ E 047°27′59.6″	688
Manokoakora (7/29/13–8/1/13)	Forest	S 21°17′10.7″ E 047°32′46.1″	644
	Forest w/odor	S 21°17′11.0″ E 047°32′47.1″	646
	Village	S 21°17′12.0″ E 047°32′36.1″	612
	Village w/odor	S 21°17′12.5″ E 047°32′40.2″	612
	Agriculture	S 21°17′17.7″ E 047°32′46.0″	605
	Agriculture w/odor	S 21°17′15.0″ E 047°32′42.5″	616
Bevohazo (8/3/13–8/6/13)	Forest	S 21°12′20.4″ E 047°30′10.0″	720
	Forest w/odor	S 21°12′21.8″ E 047°30′07.0″	689
	Village	S 21°12′37.0″ E 047°29′54.5″	616
	Village w/odor	S 21°12′30.6″ E 047°29′54.7″	616
	Agriculture	S 21°12′37.9″ E 047°29′52.0″	616
	Agriculture w/odor	S 21°12′36.1″ E 047°29′54.4″	602

Mosquitoes were collected from June through August 2013, using CDC miniature light traps (Model 512, John W. Hock Company, Gainesville, FL, USA) according to methods outlined in [14]. One of the two traps in each land-use site was randomly selected and baited with a synthetic human-derived odour, 3-Methyl-1-butanol (Fisher Scientific, Waltham, MA, USA catalog # 5001438080) [15] to improve capture of human malaria vectors, and field produced CO^2^ [14]. Each trap had a light sensor (LCS-2 Photo Switch, John W. Hock Company, Gainesville, FL, USA) attached that triggered the fan and light to turn on at sunset and off at sunrise, ensuring consistency among trapping sessions. Approximate distance of mosquito traps from the nearest livestock pen was also recorded.

Adult mosquitoes were identified morphologically on-site according to [16, 17] and stored in vials containing the desiccant Drierite (Fisher Scientific catalog # 075783B). All collected female *Anopheles* mosquitoes were dissected, and the head/thorax were separated to test for the presence of *P.falciparum*, *P. vivax*-*210* and *P. vivax*-*247* circumsporozoite proteins to determine malaria infection using the standard csELISA protocol as outlined in [18, 19]. These specific *Plasmodium* species were chosen because *P. falciparum* and *P. vivax* are the two most prevalent species of malaria in Madagascar [2].

### Statistical analysis

Data analysis was performed using SAS 10.1 ^®^ (SAS, Inc., Cary, North Carolina). General exploratory data analyses and bivariate relationships of variables of interest were examined. Poisson Regression models were used to examine the relationship between the prevalence of *Anopheles* and variables in the study that may influence mosquito prevalence. The independent variables included in the model were: land-use, village, odour, moon illumination, precipitation, temperature and elevation (Table 1). The dependent variable was *Anopheles* abundance. To examine whether land-use variability was altering overall mosquito captures or *Anopheles* specifically, the model was run to investigate the ratio of the number of *Anopheles* to the total number of mosquitoes by setting the total number of mosquitoes as the “offset” variable and *Anopheles* as the dependent variable in the model. Some data that were “missing at random” was left out of the analysis.

## Results

The total number of female *Anopheles* mosquitoes captured in 36 sites (two traps in each land-use site, agriculture, village, and forest site, in six villages) was 414. The number of *Anopheles* mosquitoes captured in agricultural sites, village sites, and forest sites was 272, 124, and 18, respectively (Table 2). The most *Anopheles* mosquitoes and known vectors were captured in the village of Manoakoakora (n = 173) (Table 3).

**Table 2:** Summary of *Anopheles* species captured, including known malaria vectors, in each land-use site in RNP, Madagacar

Land-use site	*Anopheles gambiae s.l.* ^a^	*An. funestus* ^a^	*An. mascarensis* ^a^	*An. squamosus*	*An. coustani* ^a^	*An. unknown*	Total	Other mosquitoes
Agricultural	169	61	15	0	2	25	272	610
Village	83	21	2	5	4	9	124	472
Forest	3	1	0	0	3	11	18	974

^a^Known malaria vectors

**Table 3:** Summary of *Anopheles* species captured, including known malaria vectors, in each of the six village-associated sites sampled in RNP, Madagacar

Site	*Anopheles gambiae s.l.* ^a^	*An. funestus* ^a^	*An. mascarensis* ^a^	*An. squamosus*	*An. coustani* ^a^	*An. unknown*	Total	Other mosquitoes
Ambatolahy	9	0	1	3	6	0	19	85
Ambodiaviavy	45	25	1	2	0	0	73	164
Bevohazo	27	9	2	0	0	2	40	730
Manokoakora	106	24	9	0	0	34	173	554
Menarano	68	22	3	0	3	6	102	190
Vohiparara	0	3	1	0	0	3	7	334

^a^Known malaria vectors

Five *Anopheles* species were determined morphologically on-site, four of which are recognized malaria vectors: *An. funestus s.l., An. gambiae s.l., An. mascarensis,* and a recently recognized vector, *An. coustani* [3]. *Anopheles squamosus,* a non-vector was also identified, and the remaining *Anopheles* specimens were identified to the genus level, but not species (Tables 2, 3). Most of the *Anopheles* mosquitoes captured at agricultural sites were *An. gambiae s.l.* (62.1 %, n = 169), followed by *An. funestus* (22.4 %, n = 61).

**Fig. 2:**
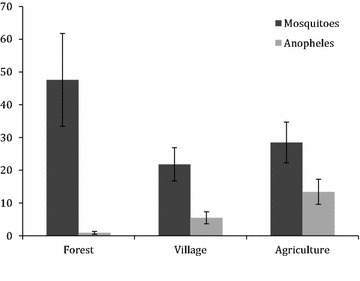
Average number of mosquitoes and *Anopheles* trapped each night by land-use type, with *standard error bars*, in six villages near Ranomafana National Park, Madagascar

**Fig. 3:**
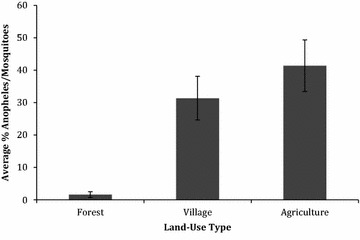
Average percent *Anopheles* of total mosquitoes trapped each night by land-use type, with *standard error bars*, in six villages near Ranomafana National Park, Madagascar

The average percent of *Anopheles* of the total number of mosquitoes trapped each night in each village were ranged from 2.8 % (SE = 1.26) in Vohiparara, to 53.7 % (SE = 12.45) in Menarano (Table 4). The average percent of *Anopheles* of the total number of mosquitoes trapped each night at each land-use site was 1.6 % (SE = 0.91) in forest sites, 31.4 % (SE = 6.74) in village sites, and 41.4 % (SE = 7.96) in agricultural sites (Figs. 2, 3).

**Table 4:** Summary and comparison of *Anopheles* and total mosquitoes trapped in each village per night in six villages near Ranomafana National Park, Madagascar

Village	# Trap nights	Average # *Anopheles* trapped per night (SE)	Average # mosquitoes trapped per night (SE)	Average % *Anopheles* among total mosquitoes trapped per night (SE)
Ambatolahy	4	7.8 (0.73)	27.3 (1.42)	28.4 (5.90)
Vohiparara	4	1.8 (0.23)	63.5 (5.02)	2.8 (1.26)
Ambodiaviavy	3	24.3 (6.21)	63.0 (9.04)	38.6 (13.20)
Menarano	4	25.5 (4.35)	47.5 (5.96)	53.7 (12.45)
Manokoakora	3	57.7 (5.39)	193.3 (12.63)	29.8 (9.00)
Bevohazo	3	13.3 (2.20)	253.3 (23.66)	5.3 (4.84)

A regression model was run with the number of captured *Anopheles* mosquitoes as the outcome, and a type 3 analysis indicates that land-use is a significant predictor (p  < 0.0001). A full Poisson regression model was run with all seven independent variables (land-use, odour, village, moon illumination, temperature, precipitation and proximity to animal pens), and the following were significant: land-use (p < 0.0001), village (p < 0.0001), odour (p < 0.0001), and proximity to livestock pens (p < 0.0001). When examining each land-use site separately, all categories (forest, village, agriculture) were significantly different from one another. The expected log counts of *Anopheles* for village and forest sites were 0.63- and 1.95-fold lower than those from agricultural sites. When examining the relationship between the ratio of *Anopheles* to total number of mosquitoes captured and the independent variables listed above, the following variables were significant: land-use (p < 0.0001), village (p < 0.0001), proximity to animals (p < 0.0001) and moon illumination (p = 0.0047). Traps located in close proximity (<3 m) to livestock pens were 2.62 times more likely (p < 0.0001) to capture Anopheles mosquitoes than those traps that were not in close proximity to animal pens.

All 414 female *Anopheles* mosquitoes were individually screened for the circumsporozoite protein using the well-established csELISA protocol [17, 18]. Three mosquitoes (0.7 % of total captured), were found positive for *P. vivax*-*210* circumsporozoite proteins. These mosquitoes were found in *An. gambiae s.l.* and *An. funestus* and trapped in three different locations: *An. funestus* in Manokoakora agricultural site, *An. gambiae s.l.* in Manokoakora village site, and *An. gambie s.l.* in Bevohazo village site.

Out of a total of 305 study participants, 272 individuals consented to malaria RDTs. 3.7 % were positive for *P. falciparum* (Table 5). No individuals tested positive for PAN *Plasmodium* (*P. vivax*, *P. ovale*, *P. malariae*). All positive individuals were from different households, and *P. falciparum* infection was detected in five of the six villages surveyed (Table 6). None of the positive individuals were febrile during the health assessments; however, several did report low-grade symptoms (i.e., body aches). Seven of the ten positive individuals were females aged: 2, 2.5, 8, 16, 23, 26, and 35 years. One female was post-partum. The three males that tested positive were 7, 9, and 21 years of age.

**Table 5:** Summary of human population demographics, nurse assessments, and ITN ownership and usage as recorded in this study

Characteristic	N (%)	Total N^a^
Demographics		
Sex		305
Female	174 (57.0)	
Male	131 (43.0)	
Age (years)		303
<5	50 (16.5)	
5 to 17	113 (37.3)	
≥18	142 (46.9)	
Nurse assesments		
BMI^b^ (<18.5)	13 (11.8)	110
Stunting^c^ (< −2 SD)	10 (20.0)	50
Underweight^2^ (< −3 SD)	17 (34.0)	50
Febrile (temp > 100.4° F)	3 (1.1)	265
Positive malaria RDT	10 (3.7)	272
Insecticide Treated Net (ITN) ownership		
Live in house with ITN	315 (94.3)	334
If yes, number of ITNs		
1	36 (11.4)	315
2	115 (36.5)	315
3	125 (39.7)	315
4	31 (9.8)	315
5	0 (0.0)	315
6	8 (2.5)	315
ITN Usage		
Never	70 (23.3)	301
Children <5	10 (3.3)	
Females ≥18	18 (6.0)	
Not every night	15 (5.0)	301
Children <5	2 (0.07)	
Females ≥18	4 (1.3)	
Every night	216 (71.8)	301
Children <5	37 (1.2)	
Females ≥18	59 (2.0)	

^a^Not all individuals were willing to participate in all components of survey and health assessments, therefore total n is listed

^b^BMI reported for the ≥18 year-old population who completed the physical assessment

^c^Stunting and underweight reported for <5 year-old population

**Table 6:** Summary of human cases of *P. falciparum* found in six villages sampled near Ranomafana National Park, Madagascar

Village	Positive *P. falciparum* (%)	Household members (n)	Malaria RDTs administered (%)
Ambatolahy	2.2 (n = 1)	56	80.3 (n = 45)
Ambodiaviavy	2.1 (n = 1)	53	71.7 (n = 38)
Bevohazo	5.1 (n = 3)	58	94.8 (n = 55)
Manokoakora	2.1 (n = 1)	52	88.5 (n = 46)
Menarano	8.5 (n = 4)	68	69.1 (n = 47)
Vohiparara	0.0 (n = 0)	47	87.2 (n = 41)

When questioned about ITN ownership, 94.3 % of individuals lived in households with ITNs (Table 5). When asked how often individuals slept under a bed net over the past four weeks (Additional file 2), 70 (23.3 %) reported never sleeping under a bed net. Of the individuals that never sleep under ITNs, 10 (14.3 %) were children under the age of 5, and 18 (25.7 %) were women over the age of 18 (Table 5).

## Discussion

In forested areas of Madagascar, land-use plays a critical role in *Anopheles* abundance, with highest *Anopheles* abundance in agricultural sites followed by village and forested sites. More malaria vectors, *An. gambiae s.l.* (62.1 %, n = 169), were trapped in agricultural sites in this study than in any other land-use site. Certain agricultural practices, such as the irrigation of rice fields in our agricultural study sites, may increase the number of breeding of mosquitoes [9], which can lead to an increase in malaria prevalence [3]. It is possible that when converting land from forests into rice cultivation sites Madagascar, the irrigation structure using rainfall to flood rice fields creates pools of stagnant water with small amounts of surface vegetation, creating ideal breeding habitats for *Anopheles* [20, 21].

Mosquito traps set within <3 m of livestock pens were significantly more likely (p < 0.0001) to capture *Anopheles* mosquitoes than those far from livestock pens. In a previous study in Madagascar, *An. gambiae* and *An. arabiensis* showed an innate preference for calf odour over human odour [22], perhaps providing an explanation for our capture numbers near livestock pens. Another study suggested that *An. arabiensis* exhibits a high degree of zoophily in Madagascar [23], which may also explain these livestock associated capture numbers. In this study, however, *An. gambiae s.l.* and *An. arabiensis* were not differentiated, so it cannot be assumed that *Anopheles* caught near livestock pens were indeed *An. arabiensis*. Even if zoophilic, *An. arabiensis,* a well-recognized malaria vector, is a threat to human health, and may pose risk to humans tending to livestock.

The large number of cattle in Madagascar may also increase the number of dead end hosts for human malaria, and therefore actually decrease overall malaria transmission in the area. This concept is known as zooprophylaxis [24]. Further research investigating the role of cattle and human *Plasmodium* spp. infection in this region will elucidate whether or not cattle ownership has potential use as a form of malaria vector control, or if it increases human exposure to malaria vectors. Whether protecting human inhabitants through zooprophylaxis, or attracting malaria mosquitoes to human inhabitants as found in [25], livestock placement and husbandry practices should be incorporated into malaria prevention and intervention strategies. Targeted insecticide control for humans as well as livestock may provide additional protection against malaria carrying mosquitoes in Madagascar.

In this study, known malaria vectors (*An. gambiae s.l.*, *An. funestus*, *An. mascariensis,* and *An. coustani*) were found to make up 90.1 % of *Anopheles* spp. captured in agricultural land-use sites during the months of June–August, suggesting that these sites may be high-risk areas for malaria. Such geographic data on parasite vectors can inform and greatly improve malaria control and elimination programmes [26].

When screening captured mosquitoes for malaria parasites, 0.7 % were positive for malaria species *P. vivax*-*210.* One of the villages sampled, Manokoakora, differed from the others in that gold extraction has become common practice in this village and its surrounding agricultural fields. The method of extraction involves digging deep holes that often remain full of still standing water, and vegetation from rice cultivation, which may create an optimal *Anopheles* habitat. This land-use modification may explain why 41.8 % (n = 173) of all *Anopheles* in this study were caught in Manokoakora (Table 3), and two of the three mosquitoes that tested positive for *P. vivax*-*210*, were captured in and around this village. While this is a low number, and may be due to a larger *Anopheles* sample size in this village, with the expansion of gold-mining practices in RNP, further research examining the significance of gold mining on mosquito-borne diseases in the region is necessary.

Unlike the *P. vivax*-*210* detected in mosquitoes, when using RDTs, only *P. falciparum* was detected in associated human populations. Historically, it was thought that erythrocyte Duffy blood group negative individuals, mainly of African ancestry, are resistant to *P. vivax* infection [27], due to the Duffy protein acting as an entrance point into erythrocytes. Previous studies have identified Duffy negative individuals in Madagascar infected with *P. vivax*, suggesting that in some regions of Madagascar, *P. vivax* is no longer dependent on the Duffy antigen for establishing human infection and disease [27]. Future work in the RNP region has the potential to reveal whether or not the populations in this region are Duffy negative and hence “resistant” to *P. vivax* infection, perhaps explaining why *P. vivax* infection was identified in mosquito vectors in this study, but not in the human populations. Other potential explanations for this discrepancy between human and mosquito infections could be due to false positives in mosquitoes [28], missed *P. vivax* human infections due to low sensitivity (possibly due to low detection level limits) of the RDT used, or that the number of people tested was so low that *P. vivax* could not be detected.

Most individuals surveyed in this study reported ownership and usage of ITNs (Table 5), which likely plays an important role in the protection of humans in the region from malaria; however, not all individuals in target demographics (children under the age of five and women over the age of 18) reported sleeping under ITNs regularly (Table 5). Since malaria has the highest fatality rates in women and children under the age of five, it is crucial to emphasize the importance of regular ITN usage in the region, especially for these individuals.

## Conclusions

By combining human health assessments and surveys with environmental vector sampling in varying land-use sites in rural Madagascar, this study revealed that malaria vectors are abundant in agricultural land-use sites, in particular in proximity to animal pens, and that humans in the region test positive for *P. falciparum* in the winter months (Jun–Aug), while *P. vivax* 210 is detected in mosquitoes, and 23.3 % of individuals report never using ITNs. A deeper understanding of the locations where malaria vectors are prevalent, such as the agricultural land-use sites or near livestock pens presented in this study, is necessary to improve targeted malaria prevention strategies in the region.

## Additional files

10.1186/s12936-016-1164-2 Individual health assessment form.
10.1186/s12936-016-1164-2 Examples of survey questions asked regarding ITN ownership and usage.
