# Supplementary material for: Small-scale land-use variability affects Anopheles spp. distribution and concomitant Plasmodium infection in humans and mosquito vectors in southeastern Madagascar
Source: Malar J. 2016 Feb 24;15:114. doi: 10.1186/s12936-016-1164-2 (PMC4779247; doi:10.1186/s12936-016-1164-2)
Supplement: Supplementary file 1 — 10.1186/s12936-016-1164-2 Individual health assessment form. [file 12936_2016_1164_MOESM1_ESM.docx]

**Additional file 1**. Individual Health Assessment Form

| **Individual Health Assessment** | |
| --- | --- |
| **Rapid Detection Tests:** | **E1:** Malaria RDT Date/time of test: _______________   1. Neg 2. Pos *P. fal* 3. Pos non-*P. fal* 4. Pos both 5. Not done   Fecal Sample Date/time of test: ________________  **E2:** Adenovirus **E3:** Rotavirus  00 Neg 00 Neg  01 Pos 01 Pos  99 Not Done 99 Not Done |

| **E5** | Weight:_______________ kg | | |  |
| --- | --- | --- | --- | --- |
| **E6** | Height: _______________ cm | | |  |
| **E7** | Temperature: __________ ^0^F | | | 00 Normal  01 >100.4  02 Not taken |
| **E8** | Resp Rate:____________/ min | | | 00 Normal  01 Abnormal  02 Not taken |
| **E9** | Heart Rate: ___________/ min | | | 00 Normal  01 Abnormal  02 Not Taken |
| **E10** | Blood Pressure: ____________ mmHg | | | 00 Normal  01 Abnormal  02 Not Taken |
|  | **Systems** | **Normal** | **Abnormal** | **Comments** |
| **F1**  **F2**  **F3**  **F4**  **F5**  **F6**  **F7**  **F8**  **F9**  **F10**  **F11** | General  Appearance  Integument  HEENT  Dental  CV  Resp  GI  GU  Neuro  Msk | 00  00  00  00  00  00  00  00  00  00  00 | 01  01  01  01  01  01  01  01  01  01  01 |  |
