# Supplementary material for: Small-scale land-use variability affects Anopheles spp. distribution and concomitant Plasmodium infection in humans and mosquito vectors in southeastern Madagascar
Source: Malar J. 2016 Feb 24;15:114. doi: 10.1186/s12936-016-1164-2 (PMC4779247; doi:10.1186/s12936-016-1164-2)
Supplement: Supplementary file 2 — 10.1186/s12936-016-1164-2 Examples of survey questions asked regarding ITN ownership and usage. [file 12936_2016_1164_MOESM2_ESM.docx]

**Additional file 2**. Examples of survey questions asked regarding ITN ownership and usage.

| How often have you slept under a bed net in the past 4 weeks? | 00 Never  01 Less than once a week  02 More than once a week, but not every night  03 Every night  99 Don’t know/Refused |
| --- | --- |
| Do you feel that your health is:  **Interviewer:** read options aloud | 01 Poor  02 Fair  03 Good  04 Excellent |
| **For Women Only** |  |
| Have you given birth in the past 2 years? | 00 No (Skip to END)  01 Yes  99 Don’t Know/Refused (Skip to END) |
| Please think back to when you were pregnant. Did you ever sleep under a bed net? | 00 No (Skip to END)  01 Yes  99 Don’t Know/Refused (Skip to END) |
| How often did you sleep under the bed net?  **Interviewer:** read options to this question aloud. | 01 Occasionally  02 Most of the time  03 All of the time  99 Don’t Know/Refused |
